# Combined analysis of the microbiome and metabolome to reveal the characteristics of saliva from different diets: a comparison among vegans, seafood-based omnivores, and red meat (beef and lamb) omnivores

**DOI:** 10.3389/fmicb.2024.1419686

**Published:** 2024-07-15

**Authors:** Shiyu Sun, Huiqiong Zhang, Linying Ye, Litao Huang, Jieyu Du, Xiaomin Liang, Xiaofeng Zhang, Jiaxing Chen, Yingping Jiang, Ling Chen

**Affiliations:** ^1^Guangdong Second Provincial General Hospital, Guangzhou, Guangdong, China; ^2^Department of Pediatrics, Guangdong Provincial People’s Hospital, Guangzhou, China; ^3^School of Forensic Medicine, Southern Medical University, Guangzhou, Guangdong Province, China

**Keywords:** forensic, saliva, dietary habits, microbiome, metabolism

## Abstract

**Introduction:**

Revealing individual characteristics is supportive for identifying individuals in forensic crime. As saliva is one of the most common biological samples used in crime scenes, it is important to make full use of the rich individual information contained in saliva. The aim of this study was to explore the application of the microbiome in forensic science by analysing differences in the salivary microbiome and metabolome of healthy individuals with different dietary habits.

**Methods:**

We performed 16S rDNA sequencing analysis based on oral saliva samples collected from 12 vegetarians, 12 seafood omnivores and 12 beef and lamb omnivores. Non-targeted metabolomics analyses were also performed based on saliva samples from healthy individuals.

**Results:**

The results showed that the dominant flora of vegetarians was dominated by *Neisseria* (belonging to the phylum Proteobacteria), while seafood omnivores and beef and lamb omnivores were dominated by *Streptococcus* (belonging to the phylum Firmicutes). NDMS-based and cluster analyses showed that vegetarian dieters were significantly differentiated from meat dieters (seafood omnivores and beef and lamb omnivores), which may be related to the fact that high-fiber diets can create a different salivary flora structure. Variants were also detected in salivary metabolic pathways, including positive correlations with Lipid metabolism, Amino acid metabolism, Carbohydrate metabolism, and Nucleotide metabolism in vegetarians, and correlations in seafood omnivores. In order to select salivary microorganisms and metabolic markers that can distinguish different dietary profiles, a random forest classifier model was constructed in this study, and the results showed that individuals with different dietary profiles could be successfully distinguished based on the core genera and metabolites such as *Streptococcus*, Histidinyl-Valine.

**Conclusion:**

Our study provides a supportive basis for the application of salivary polyomics in order to reveal the dietary characteristics of individuals for forensic investigation and crime solving.

## Introduction

Identification of personal identity information is one of the most obscure points in forensic evidence science, and how to dig out more information from the limited physical evidence has been the crucial challenge for criminal investigators to solve the case (Ohira et al., 2009). Saliva is the more common biological material used in actual forensic examinations, and is often left at the crime scene through droplet transmission such as sneezing and coughing, as well as in the form of kissing and biting (Richardson et al., 2019; Karadayi et al., 2021). Therefore, the evidence value of saliva in forensic medicine and criminal investigation is of great importance, and it is the main investigation tool for forensic investigation of crimes and sexual assault cases (Kamodyov et al., 2013).

The oral cavity is a window for communication between human body and environment, from which nutrients are imported, which in turn promotes the succession of microbial communities. Due to the presence of different microhabitats such as teeth, tongue and buccal mucosa, the oral microbial community is shaped by forces in dynamic equilibrium (salivary flow and shedding, etc.) and interactions between microorganisms and hosts (Mark Welch et al., 2020). The microorganisms that inhabit in saliva from the oral cavity account for more than 99.9% of the total oral microorganisms (Sharma et al., 2018). Microbial characteristics of saliva can be created using any saliva residues found in bite marks or lip prints, which provide details on an individual’s age, gender, personal characteristics or health status (Yadav et al., 2024). The trace of oral microorganisms collected at the crime scene can produce investigation clues of criminal and civil cases. In forensic applications, research on human saliva microbiota focuses on providing alternative methods to address issues related to postmortem inference (Adserias-Garriga et al., 2017), fluid identification (Dobay et al., 2019), individual identification, individual characteristic inference (Moon et al., 2015) and so on. For example, Leake et al. used next-generation sequencing to analyze the saliva microbiome of two individuals at four time points to characterize individual recognition potential, and the results showed that samples from the same individual would cluster together regardless of time (Leake et al., 2016). Based on the relative abundance information of saliva microbiome, Murugesan et al. found that adults under the age of 65 have lower bacterial richness and diversity than those over 65, which was of great significance for distinguishing individuals in different age groups (Murugesan et al., 2020). In addition, they also characterized that race, gender, oral health, and other factors play a certain role in the salivary microbiome.

As the above NGS analytical studies in demonstrated the potential of the salivary microbiome in forensic identification such as sex and age inference, individual identification, etc., however, based on the assessment of long-term diet and salivary microbiomes, in-depth analysis of the link with the salivary metabolome can help to further reveal individual dietary characteristics (Filippis et al., 2014). The dynamic community succession of microbial system reflects the interaction with oral cavity and environment to some extent, and methodologies has the ability to explore the interaction between metabolites and environment. These analysis tools explore whether the human body and environment can describe the external environment and lifestyle of the human body, and obtain information about dietary characteristics by analyzing the microbial and metabolic groups of saliva. In forensic cases, this helps to narrow down and target the scope of the investigation. Personal eating habits can predict the level of specific metabolites in plasma, and plasma metabolisms emphasizes personal eating habits (Chen et al., 2022). Urine is an ideal biomarker representative to study the physiological status of the whole body, and physiological changes (gender, age, diet, daily rhythm, exercise, hormone status, lifestyle, extreme environment, etc.) can be reflected in urine (Wu and Gao, 2015). With the development of technology and knowledge, the metaphoric analysis of biological body fluids (urine and plasma) has been rich, but the readily available metaphoric analysis of saliva is limited. The composition of food can be measured by metabolites (Yuliana et al., 2022), and people’s diet and nutritional composition are different in different places, which brings more biological information to the crime scene. Various diseases can be identified by detecting salivary markers (Gardner et al., 2020), but there are few studies on salivary metabolisms with eating habits.

Research has shown that changing the intake of three major constant nutrients, including carbohydrates, proteins, and fats, significantly affects the composition of the microbial community (Le Huërou-Luron et al., 2010). The human microbiome is a multidimensional and interrelated microbial circle. The microbial composition structure and competition often predict diverse information such as life diseases (Nasidze et al., 2009). However, a single microbiome analysis does not fully reflect individual characteristics, and combined multi-omics analyses of salivary microbiome and metabolome functions are used to reveal information more comprehensively about the individual characteristics of the host. It is currently recognized that the main dietary habits of inland people are carnivores and vegans, while coastal people mainly consume seafood (McEvoy et al., 2012; Meriggi et al., 2024). This paper takes the joint metaphoric analysis of the microbial composition of oral saliva as the breakthrough point to characterize the relationship between microbial composition and human life in different dietary habits.

## Results

### Sequencing data of microbial communities

The study obtained 1,827,182 optimized sequences with 774,779,370 bases in total from the saliva samples of 36 healthy adult volunteers, with an average sequence length of 424bp. All the effective sequences obtained were annotated and classified at different taxonomic levels, and ultimately 780 operational taxonomic units (OTUs) were generated, which belonged to 1 domain, 1 kingdom, 23 phyla, 50 classes, 109 orders, 187 families, 356 genera, and 599 species.

### Alpha diversity analysis

**FIGURE 1:**
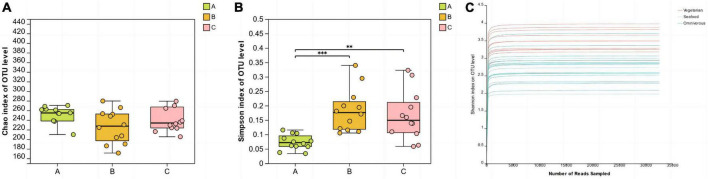
Alpha diversity analysis **(A, B)** and rarefaction curves **(C)** of the different groups. (**0.001 < *P* ≤ 0.01, ***P ≤ 0.001).

The Chao index and Simpson index were used to assess the richness and diversity of microbial communities in saliva samples. The one-way ANOVA test was employed to investigate whether there were significant differences in alpha diversity index values among groups. In this study, there were no significant differences in the Chao index among the three groups. The results of the Simpson diversity index showed that the Simpson index value of group A was the lowest, which was statistically different from groups B (*P* < 0.001) and C (*P* < 0.01). Additionally, the rarefaction curves based on the Shannon index tended to flatten out, indicating that the sequencing data volume of the samples was sufficient and reasonable (Figure 1).

### Composition of salivary microbial communities among individuals with different dietary patterns

**FIGURE 2:**
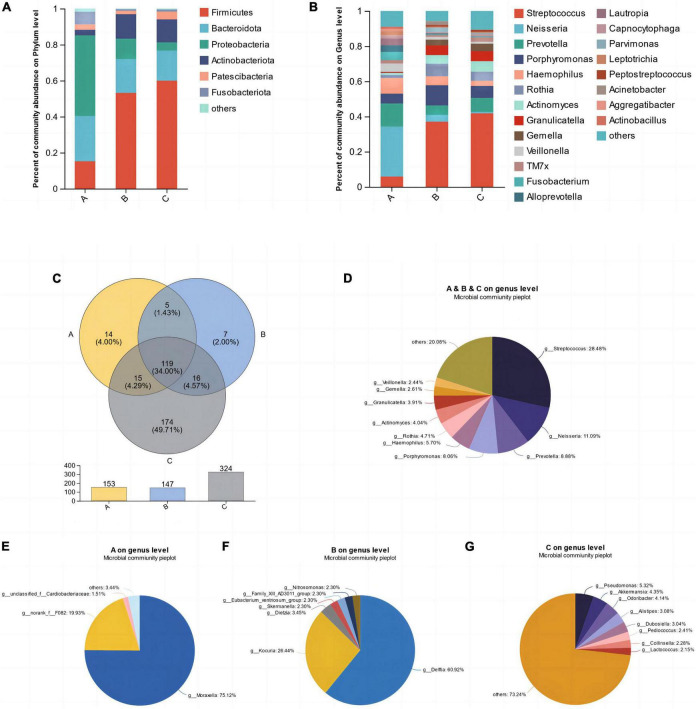
The mean relative abundances of bacterial phyla **(A)** and genera **(B)**. **(C)** Venn diagram of the different groups. **(D)** Shared microbial genera across three groups. **(E–G)** Unique microbial genera in each group.

The average relative abundance of microorganisms in three groups, A, B, and C, at the levels of phyla and genera was assessed to further reveal the characteristics of their microbial composition. As shown in the Figure 2, at the phylum level, the top five bacterial phyla with the highest relative abundance were Firmicutes, Bacteroidota, Proteobacteria, Actinobacteriota, and Patescibacteria (Figure 2A). Among them, Proteobacteria accounted for the highest proportion in group A, while Firmicutes accounted for the highest proportion in groups B and C. At the genus level, *Streptococcus*, *Neisseria*, *Prevotella*, *Porphyromonas*, and *Haemophilus* were the dominant genera in saliva samples (Figure 2B). Group A was dominated by *Neisseria* and *Prevotella*, while groups B and C were dominated by *Streptococcus*. As shown in the Venn diagram, the number of shared microbial genera among the three groups was 119, accounting for 34%, including the aforementioned dominant genera in saliva (Figure 2C). Each group also had its unique microbial genera, such as *Moraxella* in group A, *Delftia* in group B, and *Pseudomonas*, *Akkermansia*, *Odoribacter*, etc., in group C (Figures 2E–G). Among them, group C had the largest number of unique microorganisms, with up to 174 genera (Figure 2C).

**FIGURE 3:**
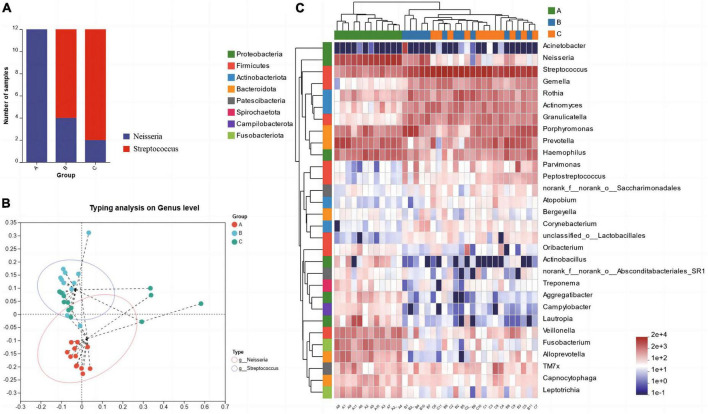
Microbiota typing analysis on genus level **(A)** and the microbiota types composition within each group **(B)**, Heatmap of the abundance of the first 30 bacterial genera and the sample clustering tree **(C)**.

The results of microbiota typing analysis revealed that the salivary samples from individuals with the three dietary patterns studied could be divided into two types based on the composition of their microbiota: type 1 and type 2, with *Neisseria* and *Streptococcus* as the top 1 species, respectively (Figure 3B). All samples from group A were classified as type 1, while most samples from groups B (66.67%) and C (83.33%) were dominated by type 2 (Figure 3A). Analysis of the heatmap of the top 30 genera in terms of total abundance at the genus level and the sample clustering tree indicated that the composition of dominant bacterial genera in groups B and C was more similar and differed from that in group A (Figure 3C).

### Overview of the oral metabolome

The original data were filtered for low-quality peaks, filled with missing values, and log transformed (log10) to reduce the errors caused by the experiment and analysis. After data preprocessing, the number of identified metabolites in positive ion mode was 1093, and the number of identified metabolites in negative ion mode was 907.

**FIGURE 4:**
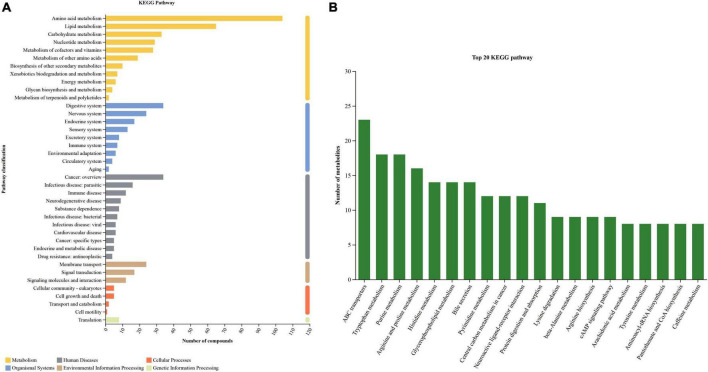
KEGG pathway classification **(A)** and top 20 KEGG pathway **(B)** for all samples.

By comparing with KEGG database, the information of metabolic pathways involved in metabolites was obtained and statistically analyzed. “Metabolism” and “Organismal Systems” were first category of the KEGG pathway, which contained the most metabolites identified above. The secondary category with the most metabolites in “metabolism” was “Amino acid metabolism”, followed by “Lipid metabolism” and “Carbohydrate metabolism”. The secondary category that contains the most metabolites in the “organism system” is the “Digestive system”. The more metabolites involved in this pathway, the more active the pathway becomes. As shown in the Figure 4, the top 20 KEGG metabolic pathways were “ABC transporters”, “Purine metabolism”, “Tryptophan metabolism” and so on.

### Using OPLS-DA to distinguish the three groups

**FIGURE 5:**
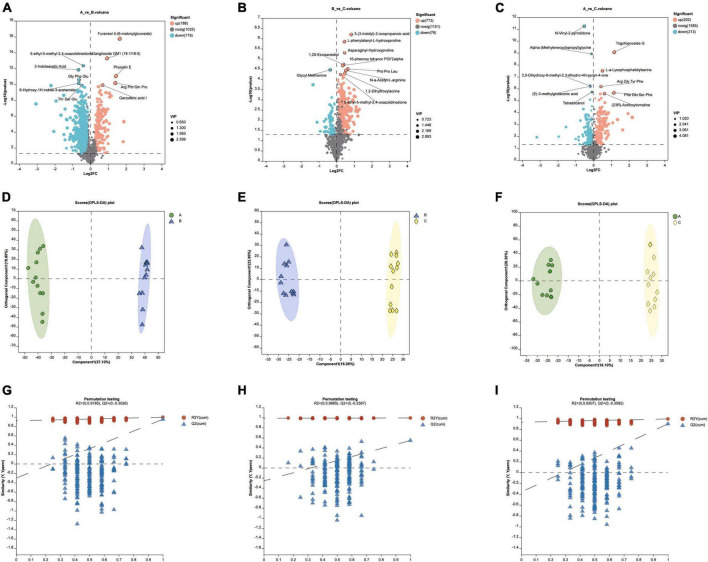
**(A–C)** Up-or down-regulated metabolites in different diet groups. **(D–F)** The Pls-DA score plot showed separation degree of the two groups (group A VS B, group B VS C, group A VS C). **(G–I)** For the validation of the above PLS-DA models, the number of random permutation tests is 200.

There were a lot of up-or down-regulated metabolites in different diet groups (Figures 5A–C). Orthogonal Partial Least Squares Discriminant Analysis (OPLS-DA) was performed to screen significant differential metabolites under the condition of VIP > 1 and *P* < 0.05 of student T test. Samples within groups were clustered, while there was a clear separation between groups (Figures 5D–F). For the reliability of the results, the OPLS-DA model was analyzed with 200 permutation tests to ensure that the model was not overfitted (Figures 5G–I).

### Identification of key modules based on WGCNA

**FIGURE 6:**
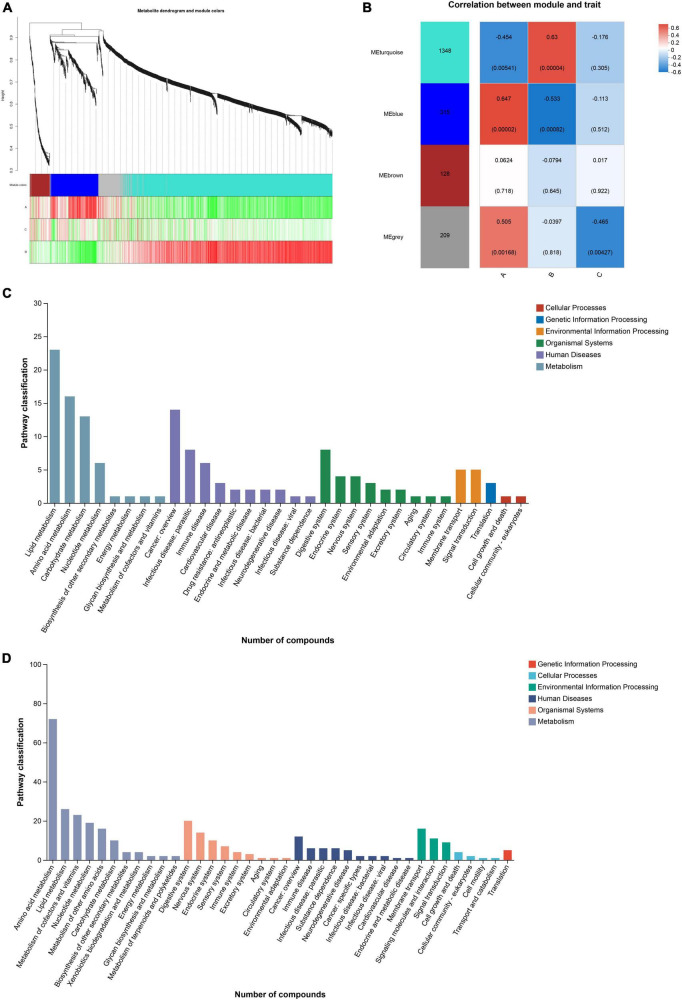
**(A)** Based on the expression trends of metabolites, metabolites are clustered into modules, where each branch represents a metabolite and each color denotes a distinct module. **(B)** The correlation between the module and the diet group is shown. **(C, D)** Pathway classification of blue and turquoise module.

The soft threshold was set to 6 and the kME value was set to 0.3 for module identification. According to the expression trend of metabolites, the metabolites were divided into three modules, and metabolites that were not divided into specific modules were classified into gray modules. The turquoise module contained 1,348 metabolites, the blue module contained 315 metabolites, and the brown module contained 128 metabolites (Figure 6A). The remaining metabolites that were not assigned to these three modules were placed in the gray module. After obtaining the modules, the correlation between the modules and diet information was analyzed by spearman analysis to explore the association between metabolic network and diet. Group A showed a positive correlation with the blue module and a negative correlation with the turquoise module, while group B exhibited the opposite pattern. Additionally, group C did not show significant correlations with the three modules mentioned above but had a negative correlation with the gray module (Figure 6B).

Functional annotation analysis of the metabolites within the modules was performed using the KEGG database. Both the blue and turquoise modules primarily belonged to the primary category of Metabolism in terms of KEGG functional classification. The secondary classification of the KEGG metabolic pathways in the blue module was mainly focused on Lipid metabolism, Amino acid metabolism, Carbohydrate metabolism, and Nucleotide metabolism. In contrast, the secondary classification of the KEGG metabolic pathways in the turquoise module encompassed Amino acid metabolism, lipid metabolism, Metabolism of cofactors and vitamins, Nucleotide metabolism, Metabolism of other amino acids, and Carbohydrate metabolism (Figures 6C, D).

### Identification of core metabolites in key modules and their correlation with microbial taxa

**FIGURE 7:**
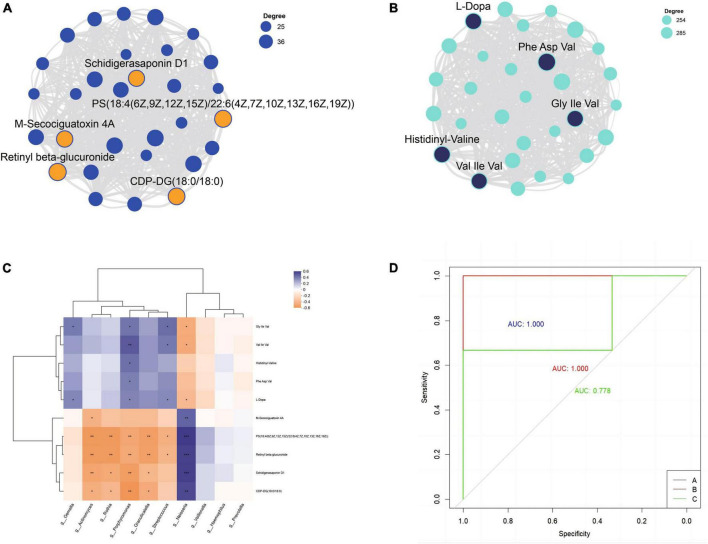
The top 5 metabolites in the blue **(A)** and turquoise **(B)** module. **(C)** Correlation analysis of core metabolites and top 10 bacterial genera. **(D)** Receiver operating characteristic curves (ROC) was used to evaluate the performance of random forest classification models; AUC indicates the area under the curve.

Each module was visualized as a network, and the top 30 nodes with the highest connectivity within each module were selected for further analysis. Based on the node degree (a measure of node connectivity, with a higher degree indicating greater importance of the node), the top five metabolites in each module were chosen as core metabolites. The top five metabolites in the blue module were Retinyl beta-glucuronide, PS[18:4(6Z,9Z,12Z,15Z)/22:6(4Z,7Z,10Z,13Z,16Z,19Z)], CDP-DG(18:0/18:0), M-Secociguatoxin 4A, and Schidigerasaponin D1 (Figure 7A). Phe Asp Val, Histidinyl-Valine, Gly Ile Val, L-Dopa and Val Ile Val were the most important metabolites in the turquoise module by visualizing the correlation between metabolites within the module (Figure 7B). By analyzing the correlation between the top 10 bacteria in relative abundance and core metabolites, *Porphyromonas*, *Streptococcus*, *Neisseria*, *Rothia*, *Granulicatella*, *Actinomyces*, *Gemella* showed significant correlations with core metabolites with higher relative abundance (Figure 7C).

### Random forest

Using the results of seven dominant bacterial genera and ten core metabolites in saliva as dependent variables, a random forest multi-class classification model was constructed based on the oral microbiome and metabolome results. The model built based on the microbiome and metabolome results performed well. The validation set (*n* = 9, with 3 samples from group A, 3 samples from group B, and 3 samples from group C) showed that 8 of the 9 validation samples were correctly classified. All samples from group A and group B were correctly classified, while 2 of the 3 samples from group C were correctly classified, and 1 was predicted to be in group B (C-6). The overall accuracy rate was 88.89%. The performance of the random forest model was evaluated through ROC analysis, and the results showed that the AUC values for groups A, B, and C were 1.000, 1.000, and 0.778, respectively (Figure 7D).

## Discussion

In this study, we conducted a joint multi-omics analysis of the salivary microbiome and metabolome of vegetarian, seafood omnivores and beef and lamb omnivores. Our study demonstrated that groups with different dietary profiles have significantly different microbiome and metabolome profiles, and that these differential profiles may have potential applications for revealing individual characteristics of forensic criminal individuals. In addition, our study identified a set of microbial and metabolite markers associated with dietary traits, showing great potential for accurately distinguishing individuals with different dietary habits.

### Comparison of salivary microbiomes

The oral cavity provides a highly heterogeneous ecological niche for microorganisms, and the composition of the salivary microbiome (Aas et al., 2005) can be influenced by both the individual and the external environment, with different dietary habits probably being the most significant influence (Stahringer et al., 2012). In our study, we found no significant difference in the species richness of salivary microbes among individuals with the three dietary profiles, whereas vegetarians had the lowest microbial diversity compared to seafood omnivores and beef and lamb omnivores. Wastyk et al. (2021) showed that high-fiber diets lead to lower microbial living habitats by examining the effects of high-fiber diets and highly fermented foods on the human immune system. Previous studies (Zaura et al., 2009) characterized the salivary microbiome to be dominated by Firmicutes, Bacteroidota, Proteobacteria, Actinobacteriota, and Patescibacteria at the facultative level, and the results of the present study showed agreement. Among them, seafood omnivores and beef and lamb omnivores had similar compositional abundance, in contrast to vegetarians who had higher Proteobacteria and lower Firmicutes. This dissimilarity was also demonstrated at the genus level, where vegetarians were dominated by Neisseria spp. (belonging to the phylum Proteobacteria), while seafood omnivores and beef and lamb omnivores were dominated by Streptococcus spp. (belonging to the phylum Firmicutes). In line with previous studies, Johansson et al. (2018) discussed the relationship between dietary milk consumption and the salivary microbiome and found that Neisseria spp. were more prevalent in the saliva of low-milk consumers, whereas a high-protein diet, i.e., a meat diet, created more Streptococcus.

In addition, studies have demonstrated that different diets create different oral ecosystems contributing to different microbial life. The present study analyzed the shared and unique microorganisms of individuals with three dietary characteristics. There were 119 microorganisms common to individuals with different diets, suggesting that core genera (Oliveira et al., 2021) such as Streptococcus, Neisseria, and Prevotella spp. comprise the salivary microbiome despite dietary differences, and Pester et al. (2010) suggest that minor low-abundance taxa may be key taxa in the ecosystem. Unique microbiomes of low fractional abundance in this study, such as Moraxella in the vegetarian group, Delftia in the seafood omnivore group, and Pseudomonas, Akkermansia, and Odoribacter in the beef and lamb omnivore group, may be important microbial markers for distinguishing between the three dietary traits. The same clustering method was applied as for the identification of enterotypes of the human gut microbiota (Arumugam et al., 2011). The results of salivary colony typing indicated that Neisseria and Streptococcus were predominant in vegetarians and carnivores (seafood omnivores and beef and lamb omnivores), respectively. Further, cluster analyses showed that carnivores could be significantly distinguished from vegetarians, whereas seafood omnivores and beef and lamb omnivores could not be distinguished due to the similarity in community composition. This may be due to the fact that seafood and beef and lamb are both high protein foods, whereas vegetarians have a predominantly fibrous diet (Laffranchi et al., 2010), and different dietary habits may result in different salivary microbiomes (Cato et al., 2023).

### Changes in the salivary metabolome

To date, there is increasing evidence (Wu et al., 2021) that microorganisms perform different functions through the metabolites they produce, and that metabolites have become an important bridge between ecological influences and microorganisms. Comprehensive multi-omics analyses of the microbiome (Fan and Pedersen, 2021) and metabolome can provide clues to correlate the microbiome with individual traits. Our metabolomics results on saliva samples showed that “metabolism” and “organismal system” contained the most metabolites, while amino acid metabolism”, “lipid metabolism” and “carbohydrate metabolism” were the most abundant metabolites in metabolism. Digestion is the most abundant metabolite in the organismal system. The top 20 metabolites in the salivary metabolic pathway include ABC transporters”, “Purine metabolism”, “Tryptophan metabolism” and others. Most of the metabolites detected in this study are consistent with previously reported metabolites in saliva of healthy individuals (Wang et al., 2014; Sridharan et al., 2019). Nutrition is known to affect oral health and metabolism in many ways (Laine et al., 2014), and in order to gain more insight into the differences in salivary metabolomes of individuals with different dietary profiles, a least squares discriminant analysis showed significant differences between vegetarians, seafood omnivores and beef and lamb omnivores. It was found that up- or down-regulation of different metabolites could distinguish different populations (Filippis et al., 2014). In addition, based on the WGCNA analysis, the key modules of different dietary characteristics could be identified, and the vegetarians showed a positive correlation with Lipid metabolism, Amino acid metabolism, etc. The seafood omnivores group, on the other hand, showed a positive correlation with Lipid metabolism. The opposite was true for the seafood omnivore group. Studies (Schmidt et al., 2016; Draper et al., 2018) that have analyzed metabolomic associations with habitual diets have shown that vegetarians tend to have higher levels of glycine.

### Individual differentiation of different dietary traits

Salivary microbiomes and metabolomics (Genco et al., 2005; Fábián et al., 2008; Aimetti et al., 2012) have been used for high-throughput identification of disease-related salivary biomarkers and to facilitate early diagnosis of a variety of diseases. We sought to correlate salivary dominant flora and core metabolites to screen for salivary microbial and metabolic markers that can be used to discriminate between populations with different dietary profiles to provide supportive evidence for forensic investigation and crime detection. Based on the network analysis, the first five metabolites in each module were screened as core metabolites, and a random forest classifier was constructed by combining the seven dominant genera in saliva. The importance ranking based on the random forest showed that the most important characteristic differences in the classification were mainly *g_Granulicatella*, *g _Neisseria*, *g_Gemella*, *g_Streptococcus*, Histidinyl-Valine. similarly, a study by Filippo et al. (2010) found that the phylum Thicket and the phylum Anopheles Mycobacterium phylum could distinguish between individuals with Western European diets and rural African diets, demonstrating that the microbiome is an important marker for discriminatory categorization. In this study, the classifier model had a recognition accuracy of 88.89%, and the AUC values indicate the high performance of the model. All the vegetarians and seafood dieters in the validation set were classified correctly, while one beef and lamb omnivore individuals were classified in the seafood omnivore group. This may be due to the fact that the beef and lamb group belongs to the same meat group as the seafood group and is associated with high fiber in the vegetarian group (Cato et al., 2023). The combination of dietary habits, individual lifestyle habits and other factors may play an important role. This requires further research to understand.

### Limitations of this study

The small sample size may lead to underrepresentation of the findings of this study. In addition, a population of three dietary profiles does not generalize to a wide variety of case scenarios, which may lead to a decrease in model accuracy when other individuals with particular dietary habits are present. Although our study revealed a functional link between the salivary microbiome and the metabolome, subsequent next steps based on more comprehensive sampling are needed; larger sample sizes, inclusion of other dietary traits, and dental cleanliness would help validate the results revealed in our study.

## Conclusion

In this study, we provided new insights into populations with different dietary profiles, using multi-omics analyses to explore salivary microbiome and metabolome profiles of vegetarians, seafood omnivores, and beef and lamb omnivores. We characterized the differences in the oral microbiomes and metabolomes of people with three different diets, identified salivary biomarkers to differentiate between the three dietary profiles, constructed a random forest classifier model to successfully differentiate between the three dietary profiles, and revealed the dietary profile of the individuals to provide supportive evidence for forensic investigation and detection of crime.

## Methods

### Sample and individual information collection

Thirty-six healthy adult volunteers aged between 18 and 56 years were recruited. These volunteers were stratified into three groups according to their dietary preferences: Group A consisted of vegans (*n* = 12), Group B comprised seafood-based omnivores (*n* = 12), and Group C included red meat-consuming omnivores (specifically those who mainly consume beef and lamb, *n* = 12). The volunteers followed the diet for at least 1 year and had no history of antibiotic use and no other gastrointestinal disorders within 3 months. The volunteers collected 2 mL saliva in the morning without brushing their teeth after fasting for 10 hours. Saliva was stored in sterile centrifuge tubes in a refrigerator at −80°C for subsequent microbial and metabolomics analysis. All participants signed informed written consent. This study was approved by the Biomedical Ethics Committee of Southern Medical University, Guangzhou, China. Before the start of this study, all individual participants included had informed consent to this study and signed a written informed consent form.

### DNA extraction, 16S rRNA gene amplicon sequencing, and data processing

Bacterial genomic DNA was extracted from saliva samples using the E.Z.N.A.^®^ soil DNA Kit (Omega Bio-tek, Norcross, GA, USA) following the manufacturer’s instructions. The concentration and purity of DNA were quantified by using an ultraviolet spectrophotometer and DNA extraction quality is checked by 1% agarose gel electrophoresis. Universal primers 338F (5′-ACTCCTACGGGAGGCAGCA-3′) and 806R (5′-GGACTACHVGGGTWTCTAAT-3′), which correspond to the bacterial 16S rRNA DNA sequence in the V3-V4 region, were used to amplify DNA samples that passed the quality inspection. PCR enrichment was performed in a 25 μl reaction with 12.5 μl 2 × Q5 Master Mix, 0.2 μM each primer, 120 ng of extracted DNA, and nuclease-free water. The PCR amplification conditions were as follows: following an initial denaturation step at 95°C for 3 min, 27 cycles were conducted which involved denaturation at 95°C for 30 s, annealing at 55°C for 30 s, and extension at 72°C for 30 s; with a final extension step at 72°C for 10 min. PCR products were purified using amplRexp beads and eluted in elution buffer. Libraries were constructed using the NEB Next UltraTM DNA library preparation kit from Illumina (New England Biolabs Inc., Ipswich, USA). Validated libraries were sequenced on the Illumina MiSeq platform (Illumina Corporation, San Diego, USA). The raw data were uploaded to the NCBI SRA database (Accession Number: PRJNA1124888).

### Metabolome sample preparation, UHPLC-MS/MS analysis and data processing

The samples were sent to the laboratory and centrifuged at 13500 r/min for 10 min at 4°C. Remove the supernatant, aliquot, and store at −80°C. At the beginning of the experiment, the samples were taken out from the −80°C refrigerator and thawed; 100 μL of the saliva sample was added to a 1.5 mL centrifuge tube containing 400 μL of a solution (acetonitrile: methanol = 1:1 (v:v)) with 0.02 mg/mL of the internal standard (l-2-chlorophenylalanine) for metabolite extraction. The mixture was vortexed for 30 seconds and sonicated at low temperature for 30 min (5°C, 40 KHz). The sample was then placed at −20°C for 30 minutes to precipitate proteins. After centrifugation for 15 min at 4°C and 13,000 *g*, the supernatant was taken off and dried with nitrogen. The sample was then redissolved in 100 μL of a solution (acetonitrile: water = 1:1) and extracted by low-temperature sonication for 5 min (5°C, 40 KHz). After centrifugation at 13000 g and 4°C for 10 min, the supernatant was transferred to a sample bottle for LC-MS/MS analysis. Simultaneously, 10 μL of the supernatant from each sample was mixed to generate a quality control (QC) sample. The QC sample was processed and tested in the same manner as the analytical samples, serving as a representative of the entire sample set and regularly injected (every 5–15 samples) to monitor the stability of the analysis.

The LC-MS/MS analysis of sample was conducted on a Thermo UHPLC-Q Exactive HF-X system equipped with ACQUITY HSS T3 column (100 mm × 2.1 mm i.d., 1.8 μm; Waters, USA). The mobile phases consisted of 0.1% formic acid in water:acetonitrile (95:5, v/v) (solvent A) and 0.1% formic acid in acetonitrile:isopropanol:water (47.5:47.5, v/v) (solvent B). The flow rate was 0.40 mL/min and the column temperature was 40°C. The mass spectrometric data were collected using a Thermo UHPLC-Q Exactive HF-X Mass Spectrometer equipped with an electrospray ionization (ESI) source operating in positive mode and negative mode. The optimal conditions were set as followed: source temperature at 425°C; sheath gas flow rate at 50 arb; Aux gas flow rate at 13 arb; ion-spray voltage floating (ISVF) at −3500V in negative mode and 3500V in positive mode, respectively; Normalized collision energy, 20–40–60V rolling for MS/MS. Full MS resolution was 60,000, and MS/MS resolution was 7500. Data acquisition was performed with the Data Dependent Acquisition (DDA) mode. The detection was carried out over a mass range of 70–1050 m/z. The raw data for the metabolome of this study have been uploaded to MetaboLights (Number: MTBLS10471).

### Statistical analysis

FLASH software (Magoc and Salzberg, 2011) was used to merge and splice the sequences, which were then assembled into Tags based on the overlap relationship between reads. Using Uparse (Version 7.1),^1^ non-redundant sequence fragments (Tags) were clustered into OTUs based on a sequence similarity greater than 97%. Chimera sequences were then identified and removed using UCHIME software (Rognes et al., 2016). RDP classifier software (Wang et al., 2007) was employed for species annotation, with a comparison threshold set at 70%. Each sequence information was compared with the SILVA biological database (silva138/16S_bacteria) for analysis. Alpha diversity is used to analyze the species diversity in the sample, using mothur (Schloss et al., 2009) (Version 1.39.5) software to calculate 3 indicators, including Chao, Shannon and Simpson. Nonmetric Multidimensional Scaling (NMDS) was used to show how dissimilarity changed between groups, calculated using QIIME (Version 1.80) software. Use LEfSe (Segata et al., 2011) (LDA Effect Size) (Version 1.0) to calculate the LDA score value. The significant flora must meet the threshold *p* < 0.05 and the LDA score value ≥ 2.0 (or ≤ −2.0).

The pretreatment of LC/MS raw data was imported into Progenesis QI software (Paglia and Astarita, 2017) (Waters Corporation, Milford, USA) for baseline filtering, peak identification, integration, retention time correction, and peak alignment. Ultimately, a three-dimensional data matrix containing sample information, metabolite name and mass spectral response intensity was obtained. The data matrix was further filtered and at least 80% of the metabolic features detected in any set of samples were retained. For specific samples with metabolite levels below the lower limit of quantification, the minimum metabolite value was estimated, and each metabolic signature was normalized to the sum. To reduce the errors caused by sample preparation and instrument instability, the response intensities of the sample mass spectrometry peaks were normalized using the sum normalization method, to obtain the normalized data matrix. Meanwhile, the variables of QC samples with relative standard deviation (RSD) > 30% were excluded and log10 logarithmic zed, to obtain the final data matrix for subsequent analysis. At the same time, the metabolites were identified by searching database, and the main databases were the HMDB,^2^ Metlin.^3^

The R package “ropls” (Version 1.6.2) was used to perform Partial Least Squares Discriminant Analysis (PLS-DA), and the number of random permutation tests was set to 200. Fold Change Analysis (FC Analysis) was used to evaluate the up-regulation or down-regulation patterns of different metabolites. The metabolites with VIP > 1, *p* < 0.05 were determined as significantly different metabolites based on the Variable importance in the projeciton (VIP) obtained by the OPLS-DA model and the p-value generated by student’s t test. Differential metabolites among two groups were mapped into their biochemical pathways through metabolic enrichment and pathway analysis based on KEGG database.^4^ Python packages “scipy.stats”^5^ was used to perform enrichment analysis to obtain the most relevant biological pathways for experimental treatments. Spearman rank correlation test was performed to assess the association between key microbiota and key metabolites. The correlation coefficient (*r*) ranges from −1 to 1, where a positive correlation represents *r* > 0 and a negative correlation represents *r* < 0.

### Machine learning process

Random forests were used to analyze and predict populations with different dietary structures. Random Forest is a classifier containing multiple decision trees, and its classification results are judged on different decision trees according to the attributes of each dimension of the test sample, and the final classification is given after comprehensively considering all the judgment results, and the maximum probability value is taken for the classification problem results, and the species class (biomarker) that is most important for sample classification is efficiently and quickly selected (Song et al., 2021). Random forest analysis was performed using the R package “randomForest” (v4.6.14) and visualized using the “pROC” package (Version 1.18.0) and the “ggplot2” package (v3.3.3). For the input features, the classifiers contained only key microbiota and metabolites. A total of 70% of the samples (*n* = 27) were randomly selected as the training set to construct the decision tree, and the remaining samples (*n* = 9) were used as the test set to verify the decision tree. The receiver operating characteristic (ROC) curve was used to evaluate the constructed model, and the area under the ROC curve (AUC) was used to represent the ROC effect to evaluate the efficacy of salivary microbial markers and significantly different metabolites in predicting different dietary structures.

## Data availability statement

The raw data for microbiome sequencing were uploaded to the NCBI SRA database (Accession Number: PRJNA1124888, https://www.ncbi.nlm.nih.gov/bioproject/PRJNA1124888). The raw data for the metabolome of this study have been uploaded to MetaboLights (Number: MTBLS10471, www.ebi.ac.uk/metabolights/MTBLS10471).

## Ethics statement

The studies involving humans were approved by the Biomedical Ethics Committee of Southern Medical University. The studies were conducted in accordance with the local legislation and institutional requirements. The participants provided their written informed consent to participate in this study.

## Author contributions

SS: Writing−original draft, Conceptualization, Investigation, Methodology. HZ: Software, Validation, Visualization, Writing−original draft. LY: Data curation, Resources, Validation, Writing−original draft. LH: Data curation, Resources, Validation, Writing−original draft. JD: Data curation, Resources, Validation, Writing−original draft. XL: Data curation, Resources, Validation, Writing−original draft. XZ: Data curation, Resources, Validation, Writing−original draft. JC: Data curation, Resources, Validation, Writing−original draft. YJ: Supervision, Writing−review and editing. LC: Supervision, Writing−review and editing.

## Footnotes

1
http://drive5.com/uparse/

2
http://www.hmdb.ca/

3
https://metlin.scripps.edu/

4
http://www.genome.jp/kegg/

5
https://docs.scipy.org/doc/scipy/
